# Isolation and Characterization of Plant Growth-Promoting Endophytic Fungi from the Roots of *Dendrobium moniliforme*

**DOI:** 10.3390/plants8010005

**Published:** 2018-12-28

**Authors:** Sujit Shah, Roshani Shrestha, Sabitri Maharjan, Marc-Andre Selosse, Bijaya Pant

**Affiliations:** 1 Central Department of Botany, Tribhuvan University, Kathmandu 2642, Nepal; sujitaug16shah@gmail.com (S.S.); rosstha371@gmail.com (R.S.); maharjansabitri89@gmail.com (S.M.); 2 Institut de Systématique, Évolution, Biodiversité (UMR 7205—CNRS, MNHN, UPMC, EPHE), Muséum National d’Histoire naturelle, Sorbonne Universités, 57 rue Cuvier, 75005 Paris, France; ma.selosse@wanadoo.fr; 3 Department of Plant Taxonomy and Nature Conservation, University of Gdansk, Wita Stwosza 59, 80-308 Gdansk, Poland

**Keywords:** orchid, *Dendrobium moniliforme*, endophytes, elicitor, indole acetic acid

## Abstract

The present study aims to identify the diverse endophytic fungi residing in the roots of *Dendrobium moniliforme* and their role in plant growth and development. Nine endophytic fungi were isolated from the root sections and characterized by molecular technique. Quantification of the indole acetic acid (IAA) compound by these endophytes was done. Further, Chemical profiling of R11 and R13 fungi was done by Gas Chromatography-Mass Spectroscopy (GC-MS). Asymbiotic seed derived protocorms of *Rhynchostylis retusa* was used for the plant growth assay to investigate the growth promoting activities of the fungal elicitor prepared from the isolated fungi from *D. moniliforme*. Among the isolated fungi, the relative dominant fungus was *Fusarium* sp. The R13 and R6 fungi were identified only at the genus level which concludes the fungi are of new species or strain. The indole acetic acid production was relatively higher in R10. Bioactive compound diversity was observed in the organic extract of R11 and R6. The presence of phenolic compound and essential oil suggest their contribution for the antimicrobial and antioxidant properties to their host plant, *D. moniliforme*. The plant growth assay result concluded, the fungal elicitor prepared from R10, *Colletotrichum alatae* was the best among all other for the plant growth activities.

## 1. Introduction

Orchids are well known for their ornamental and medicinal values, as well as for their features as indicators of ecological integrity. However, their microbial association is poorly understood and described. Orchid seeds lack endosperm, and they depend on fungi for their germination and carbon supply [1]; these fungi later become mycorrhizal, i.e., associated to their roots. Orchids also associate with endophytic fungi throughout their life cycle for their growth and development: endophytes grow in living plant tissues (biotrophy) without causing obvious symptoms or morphological modifications (e.g., no mycorrhiza), with variable impacts on host nutrition [2,3]. From germination onwards, a diverse set of fungi, which will not necessarily be mycorrhizal later, exist in orchids [4]; they belong to the phyla Basidiomycota and Ascomycota [5]. Moreover, the limit between endophytic and truly mycorrhizal fungi is difficult to delineate [6,7]. These endophytic fungi may directly or indirectly contribute to the fitness of the plants. They increase the seed germination rate, seed elongation, and the potential of roots to solubilize nutrients for uptake [8]. *Fusarium* strains have been known since the pioneering works of Bernard (1900) to improve seed germination, although they are not mycorrhizal [9], and they add to the production of secondary metabolites, which are beneficial to the host plant as well [10]. Endophytes also contribute significantly to the production of plant growth hormones, such as auxin and cytokinin, as well as in other products such as phosphatases that also contribute to plant growth [11].

*Dendrobium* is the second largest genus of the family Orchidaceae. With over 1100 species, *Dendrobium* is widely spread across the world [12]. Thirty species are reported to date from Nepal. Over the years, they have been exploited, and most of the *Dendrobium* species are now listed as endangered and threatened [13,14].

*Dendrobium moniliforme*, distributed in the central hills of Nepal, is commonly known as White *Dendrobium*. It is a typical epiphytic, a deciduous orchid species that requires less water and nutrients to grow. It has distinguishing features such as flowers with white to yellow blotch bases in their lip, and a semispherical anther cap, it and blooms April to May [15,16]. Both its ornamental and medicinal properties make *D. moniliforme* economically important [16]. The plant extracts of this species have anti-microbial and anti-oxidant activities, as well as the potential to cure osteoporosis [17]. A pending challenge is to investigate the role of endophytes on plant growth and fitness in their native environment. The current investigation aims at exploring the axenically cultivable endophytic fungi present in host root segments, and provides preliminary characterization of their potential roles in plant growth and development. Furthermore, it investigates how diversified or abundant these fungi are in synthetic media after isolation. The present research sheds new light on the biochemical properties of the fungi that may directly or indirectly help with the growth and development of the plant in its native environment. This may ultimately add to our understanding of how these endophytes can benefit the in-vitro micropropagation of orchid species.

## 2. Results

### 2.1. Molecular Identification of Endophytic Fungi

**Figure 1:**
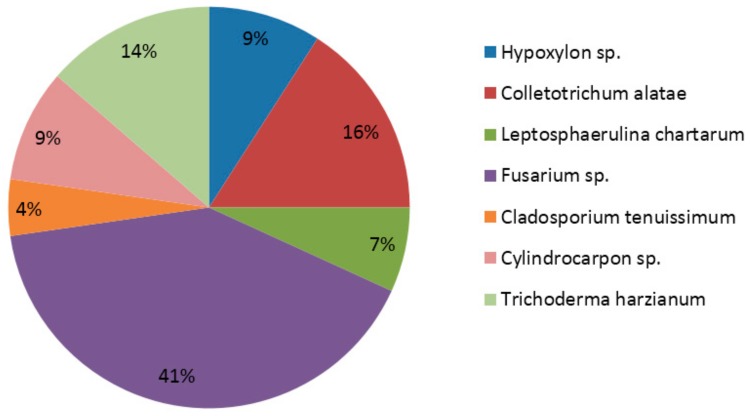
Relative abundance of endophytic fungi after isolation in synthetic media. *Fusarium* is the dominant genus in synthetic media isolated from the root of *Dendrobium moniliforme*.

A total of nine morphotypes (out of 50 isolated strains) were recovered in the isolation procedure from root pieces (Table 1). After molecular analysis, all morphotypes looked homogeneous in terms of internal transcribed spacer (ITS) sequence and all belonged to Ascomycota (Table 1), in the genera *Cladosporium*, *Colletotrichum*, *Cylindrocarpon*, *Fusarium*, *Hypoxylon*, *Leptosphaerulina* and *Trichoderma* (Table 1). Among the 50 fungi isolated from the root of *D. moniliforme*, *Fusarium* spp. was dominant (Figure 1). However, some species such as R6 and R13 showed less than 90% identity with KM013463.1- and KM268690.1-retrieved sequences from the NCBI GenBank database, respectively (Table 1). These endophytic fungi, R6 and R13, were characterized as *Hypoxylon* sp. and *Fusarium* sp. respectively, at the genus level.

### 2.2. Quantification of Indole Acetic Acid (IAA) Synthesis by Isolated Fungi

**Figure 2:**
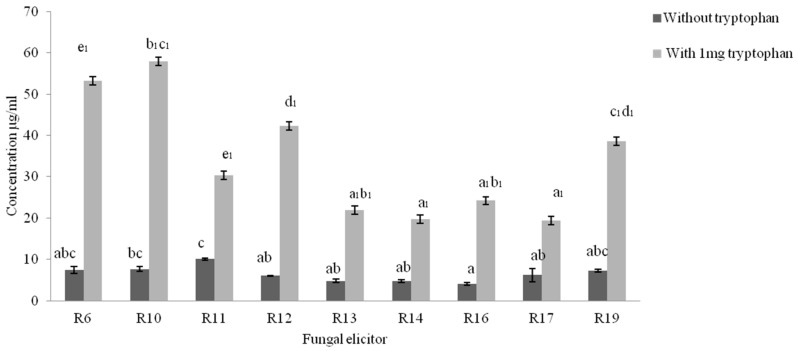
Auxin synthesis by endophytes with and without induction of tryptophan added. R10 showed relatively higher concentration of indole acetic acid (IAA) among all other isolates. The experiment was repeated three times per treatment. The bar represents mean ± SE (n = 3). Values with different letters are significantly different at *p* ≤ 0.05(Tukey test).

Indole acetic acid in culture medium was estimated by Salkowski reagent [18], and it revealed variable concentrations of IAA in the culture extracts. The indole acetic acid concentration was higher in broths supplemented with tryptophan for all endophytes when compared to cultures in broth without tryptophan. Among all these, the indole compound concentration was higher in R10 (tryptophan containing broth), while R11 showed the highest concentration of the indole acetic acid of cultures without tryptophan supplementation (Figure 2).

### 2.3. Detection of Bioactive Compound by Gas Chromatography-Mass Spectroscopy (GC-MS) analysis 

We performed GC-MS analysis to investigate the bioactive compounds of the methanol extract of R11 and R13 isolates (Table 2). Potentially bioactive compounds and essential oils were identified that may have a role in plant–fungus communication. We detected phenol 2,4-bis (1,1-dimethylethyl), 3-octadecene, hexadecanoic acid methyl ester, 3-eicosene, and octadecanoic acid methyl ester in both extracts of R11 and R13 isolates. Traces of an indole compound were detected in the extract of R11, whereas an ascorbic acid moiety was detected in extract of R13.

### 2.4. Plant Growth Assay with Fungal Elicitor Treatment

**Figure 3:**
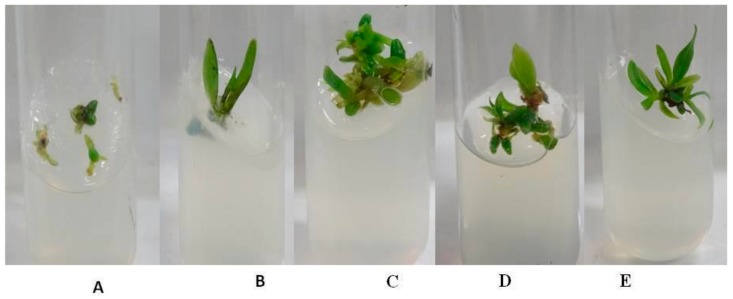
In vitro plant growth assay of *R. retusa* grown (**A**) on Murashige & Skoog (MS) media (control), or in MS media supplemented with fungal elicitors: (**B**) R6, (**C**) R11, (**D**) R10, and (**E**) R13.

**Figure 4:**
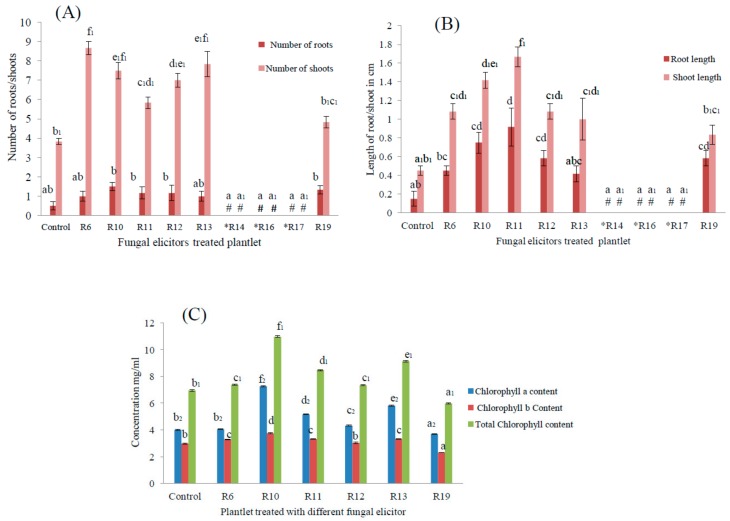
Physiological status of *R. retusa* plantlets supplemented with different fungal elicitors (n = 6 replicates per treatments). (**A**) Number of roots and shoots; (**B**) Mean root and shoot length; (**C**) quantification of total chlorophyll content. The bar represents mean ± SE (n = 6). Symbol ‘#’ denotes no growth was observed for media supplemented with fungal elicitor *R14, *R16 and* R17 Values with different letters are significantly different at *p* ≤ 0.05 (Tukey test).

The protocorms of *Rhynchostylis retusa* were grown in-vitro on Murashige & Skoog (MS) media with the supplementation of a 10% fungal elicitor solution. The growth was compared with protocorms grown on basal media (control). Protocorms supplemented with R14, R16, and R17 fungal elicitors showed poor growth and did not survive. There was significant increase (*p* < 0.05) in the growth of the plantlet after for 60 days on the media supplemented with R6, R11, R10, R13, R12. and R19 with fungal elicitors (Figure 3). Significant differences in the numbers and lengths of roots and shoots were observed. The media supplemented with a fungal elicitor from R6 and R13 showed the highest number of shoots and roots, (Figure 4A). Meanwhile, the media supplemented with fungal elicitor from R11 showed the strongest increase in root and shoot length (Figure 4B). The quantification of chlorophyll contents revealed that plantlets treated with fungal elicitor R10 showed higher total chlorophyll contents (10.96 mg L^−1^), whereas plantlets treated with the fungal elicitor R19 showed least the chlorophyll content.

## 3. Discussion

*Dendrobium moniliforme* is known for its ornamental and medicinal properties, but is considerably under the threat of extinction due to habitat loss and illegal trade [17]. Ex-situ conservation by plant tissue culture techniques is an alternative way to restore the species or support the existing population. This technique should be modified or optimized according to the need for plant growth and development. This study reveals that a diverse population of non-mycorrhizal endophytic fungi inside the root system may contribute to the in vitro growth of the plant. These fungi can have both plant growth promoting activity as well as affecting the production of secondary metabolites [19].

We isolated and identified fungi from the roots of the healthy *D. moniliforme* plants. Most of the fungi were ascomycetous. The dominant genus and species in the synthetic media was *Fusarium* sp. Although most of the *Fusarium* species are pathogenic, some can exist as symptomless endophytes [20]. Similarly, *Colletotrichum* sp. and *Leptosphaerulina* (endophytic in Asian orchids) are reported to be phytopathogens, as well as symptomless endophytes [7,21]. *Hypoxylon* were also found which belongs to Xylariaceae, which are saprobes with huge endophytic potential [22]. Both *Cladosporium* sp. and *Trichoderma harzianum* are known as endophytes and some may have plant-protective effects [23,24].

All these fungi were able to increase the concentration of indole compounds or its derivatives in our assays. Most of the fungi were able to synthesize auxin, both with and without tryptophan-containing media. However, concentrations increased two- to three-fold when the medium was supplemented with L-tryptophan. The highest auxin concentration was detected in the R10 strain when supplemented with L-tryptophan. Further assessment of the biochemical composition of the two fungi R11 and R13 by a GC-MS technique revealed the presence of an indole compound in R11 extract, while phenolic derivatives and derivatives of benzoic acid were found in both extracts, whereas ascorbic acid was only found in R13. Evidence of the indole compound, dibutyl phthalate, essential oils, and phenolic compound suggest that these compounds are required for the establishment of microbial associations with the roots of the host plant. Eicosanes may have antimicrobial and antifungal activities [25,26,27,28,29,30]. Compounds that were identified from R11 and R13 strains had the most significant effects on plant growth and development. These compounds are strong candidate for future studies, further exploring the observed growth phenotypes. Detection of an ascorbic acid derivative in the R13 stains extract (Table 2) may also have a significant role in plant growth and development [31,32]. Most importantly, some of the fungal elicitors showed significant growth-promoting effects on the protocorms stage of *R. retusa* that developed into a plantlet. Elicitors prepared from the R10, R11, R13, and R19 strain showed the most significant effects on the development of the protocorms into a plantlet with a higher growth rate, and a significantly higher number of roots and shoots, as well as increased root/shoot length when compared to the controls. Higher chlorophyll contents in plantlets treated with R10, R11, and R13 also supported their favorable roles on protocorm growth and development. However, contrasting effect of the fungal elicitors R17, R14, and R16 resulted in poor growth and the death of the protocorms. However, we have no indication as to what caused this higher toxicity leading to the death of protocorms treated with elicitors from strains R14, R16, and R17.

The positive effect of the *Fusarium* strains in the present investigation is well supported by Vujanovic and Vujanovic [8], introducing the concept of ‘mycovitalism’ based on the effect of a strain of *Fusarium semitectum* on orchid seed germination. Therefore, the isolated fungi may directly or indirectly contribute to the fitness of the orchid species in the native environment. One may be surprised that fungi produce the compounds that are vital to early development of orchids. Recent research tends to indicates that the plants are holobionts [33] and that there is increasing evidence that plants are dependent on the microbes inhabiting inside them [34]. In vitro axenic growth may have to re-introduce these compounds, since the rich naturally surrounding mycoflora that are normally present in soils, is missing.

## 4. Materials and Methods

**Figure 5:**
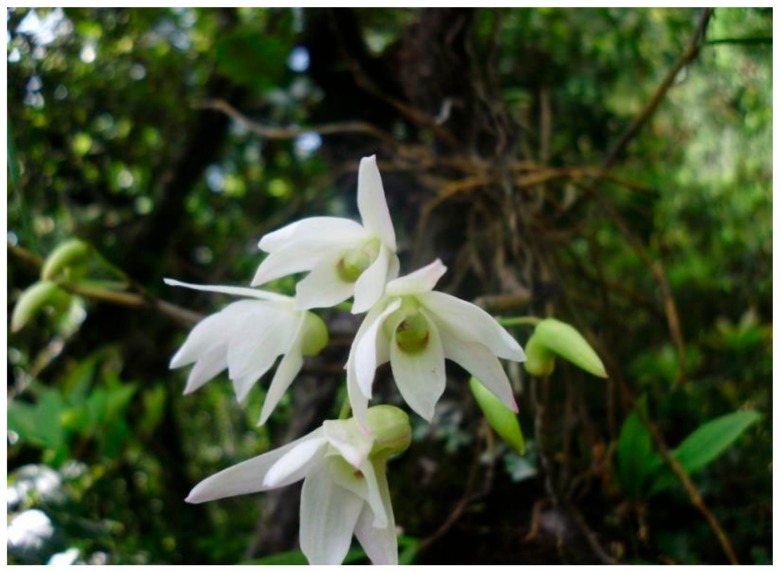
*Dendrobium moniliforme* in its natural epiphytic habitat with typical white flowers with a yellow blotch at the base of the lip, at Makwanpur district, Daman community forest.

Root sections from different individuals of epiphytic *D. moniliforme*, growing on *Quercus semecarpifolia* as their host tree (Figure 5), were collected from the community forest of Daman, Makwanpur District, situated at 27°36′29″ N, 85°5′39″ E at 1500–2500 m height in the central hills of Nepal in the month of May (blooming period). The root section was cut with a sterile blade, stored in a zipper plastic bag, and swiftly brought to the Central Department of Botany, Plant Biotechnology Research Laboratory, Kathmandu, Nepal for further research. Roots sections were collected without harming the plant population. After the collection, roots were store at 4 °C for 12 h before the isolation of fungi.

### 4.1. Surface Sterilization of Roots

Surface sterilization of root sections was done according to the protocol by Khan et al. [35]. Roots were treated for surface sterilization in order to remove epiphytic and surface-adhering microbes. Roots were washed with Tween-20, and then rinsed in running tap water for 20 min before the aseptic sterilization process was carried out in a laminar hood. Here, eight roots sections were treated with 75% ethanol for 1 min, followed by 3% sodium hypochloride (NaOCl) for 10 min, and 90% ethanol for 30 s. Finally, sections were rinsed with sterile water three times and cut into 2 cm long sections with a sterile scalpel.

### 4.2. Endophytes Isolation and Axenically Culture

To isolate endophytes two pieces of 2 cm length root sections were placed in potato dextrose agar (PDA) plates supplemented with 50 μg mL^−1^ chloramphenicol incubated at 25 °C for seven days. A total of six plates were prepared. The growth of various fungi was observed, and they were further screened according to their colony pattern, and sub-cultured axenically in freshly prepared PDA plates. The resulting unique pure cultures were preserved for identification at 4 °C.

### 4.3. DNA Extraction, PCR Amplification and Sequencing

The mycelia of individual isolates were stored in separate sterile cryovials in a 50% glycerol and 5% saline water solution at 4 °C until DNA extraction. The samples were made free from glycerol stock and revived on PDA plates before DNA extraction. The fungal mycelia of 300 mg was used for DNA extraction. The Fungal genomic DNA isolates were extracted by the CTAB method and PCR amplification of the ITS region of nuclear ribosomal DNA (rDNA) was done using primer ITS1-F and ITS4 [36]. The PCR conditions were programmed as follows: one cycle of 4 min at 95 °C, followed by 35 cycles at 95 °C for 30 s, 53 °C for 30 s, 72 °C for 1 min 30 s, and extension at 72 °C for 7 min. Negative controls (absence of DNA template) were used to detect DNA contamination. The forward and reverse sequence was established as in Séne et al. [36], and a consensus sequences were obtained by using the BioEdit tool (version 7.0.8). The sequence of one representative strain per morphotype was deposited in the GenBank data base. These sequences were compared with other available ITS sequences of related fungi using the NCBI BLAST tool online (https://blast.ncbi.nlm.nih.gov/Blast.cgi).

### 4.4. Biochemical Assays

Biochemical assays for each fungal isolate were performed to estimate the concentration of auxin (indole acetic acid, IAA).

### 4.5. Quantitative Estimation of IAA in Culture Extract

Fungal isolates were grown in 20 mL czapek dox agar (CDA) medium (pH 6.5), with or without a 1 mg supplement of L-tryptophan [36]. The inoculated broths were incubated in shaker incubator at 25 °C, 120 rpm for 10 days. After incubation, they were centrifuged at 12,000 rpm for 10 min at 4 °C. To determine the production of IAA, one mL of supernatant was mixed with 2 mL of Salknowski reagent [18], and incubated in dark for 30 min. After the appearance of a pink color, the optic density was measured at 530 nm using an UV-VIS spectrophotometer (ChromTech-CT 8200). The IAA concentration of the extract was quantified against separately prepared standard IAA curves (10 to 100 µg mL^−1^). Three biological replicates were performed for each experiment.

### 4.6. Identification of Compounds Present in Fungal Extract

Fungi were grown in 300 mL czapek dox broth (CDB) for 15 days. The broth was then centrifuged at 10,000 rpm for 30 min and the supernatant was filtered on Whatman filter paper Grade 1:11 μm (medium flow filter paper). The pH of the filtrate was adjusted to 2.5 by 1 N HCl. Then, an equal volume of HPLC-grade ethyl acetate (Fisher Scientific, Mumbai, India) was added to the filtrate, shaken, and collected by using a separation funnel. This was repeated three times. The ethyl acetate extract was then subjected to a rotary evaporator, (DLAB: RE100-Pro) and it was dried at 40 °C. The residue left over was then re-suspended in 2 mL of HPLC-grade methanol (Fisher Scientific, Mumbai, India). GC-MS analysis of the methanolic extract was done on a GCMS-QP2010 Ultra (Shimadzu Europa GmbH, Dusseldorf, Germany) instrument fitted with RTX-5MS (30 × 0.25 × 0.10 m) column. The initial temperature of the instrument was set to 100 °C for 1 min, with increased at the rate of 3 °C min^−1^; the end temperature of the oven was increased to 250 °C. The helium flow rate of the instrument was 1 mL min^−1^ with an ionization voltage set at 0.80 KV. Samples of 1 µL were injected in splitless mode. The mass spectral scan range was set at 30 to 600 (*m*/*z*). Identification of potentially bioactive compounds present in the extract done by comparison to the library of National Institute of Standard and Technology, NIST, US.

### 4.7. Plant Growth Assay after Supplementation with Fungal Elicitor

A fungal elicitor solution was prepared from the supernatant of 10-days old fungus inoculated in czapek broth medium. The entire broth was centrifuged at 5000 rpm, and the supernatant was filtered through Whatman filter paper Grade 1:11 μm (medium flow filter paper). The flow-through was used as the fungal elicitor solution. MS media supplemented with fungal elicitor was prepared (9:1) [37]. The media was than autoclaved 121 °C for 20 min [38]. The protocorms of *R. retusa* were derived from the asymbiotically germinated seed (30 days old) on MS media. For the plant growth assay, six biologically independent replicates were used. Protocorms grown on MS were supplemented with fungal elicitor and observed for 60 days. Growth pattern of the plantlets were recorded after 60 days. The plantlets were grown in aseptic conditions for 2 months under a 16 h photoperiod, and at 25 ± 2 °C. MS basal medium was taken as the control for the plant growth assay

### 4.8. Quantification of Chlorophyll Content in Fungal Elicitor Plantlet

After 65 days of growth, plantlets that were subjected to the quantification of chlorophyll content. Plantlets were crushed in 80% acetone and centrifuged at 5000 rpm for 30 min. The supernatant was filtered and its absorbance was measured at 663 nm and 645 nm. Chlorophyll content was calculated by using following formula, where A_663_ and A_645_ is the absorbance at 663 nm and 645 nm, respectively [39], and expressed in mg L^−1^.

Chlorophyll *a* content = 12.7(A_663_) − 2.69(A_645_)

Chlorophyll *b* content = 22.9(A_645_) − 4.68(A_663_)

Total chlorophyll content = 20.2(A_645_) + 8.02(A_663_)

### 4.9. Statistical Data Analysis

Estimation of IAA was done independently for three replicates, and plant growth assays were performed independently for six replicates to measure the shoot and root length. Both were tested by one-way ANOVA with the alpha error level set at *p* ≤ 0.01 (Tukey HSD test).

## 5. Conclusions

The present investigation reveals several endophytes residing in the roots of the orchid *D. moniliforme* by isolation and molecular characterization. Fungal elicitors prepared from these orchid endophytes may be used in the future as a tonic for the in-vitro mass propagation of orchids, and some of the compounds identified in this study may be investigated for their impact on orchid germination.

## Figures and Tables

**Table 1:** Molecular identification and GenBank accession numbers of endophytic fungi isolated from the roots of *D. moniliforme*.

Morphotype	Tentative Affiliation	No. Isolates	Query Coverage	Percentage of Identity	Accession Code
R6	*Hypoxylon* sp.	4	99%	88%	MH532318
R10	*Colletotrichum alatae*	7	100%	100%	MH532312
R11	*Leptosphaerulina chartarum*	3	100%	100%	MH532310
R12	*Fusarium* sp.	6	100%	100%	MH532313
R13	*Fusarium* sp.	7	100%	84%	MH532317
R14	*Cladosporium tenuissimum*	2	100%	100%	MH532316
R16	*Fusarium equiseti*	5	100%	100%	MH532315
R17	*Cylindrocarpon* sp.	4	95%	100%	MH532314
R19	*Trichoderma harzianum*	6	100%	100%	MH532311

**Table 2:** List of the various bioactive compounds identified from R11 and R13 organic fungal extracts by GC-MS analysis.

Peak	Retention Time	Area%	Compound Identified	Fungal Extract	Base *m*/*z*
1	5.381	1.24	7-Methyl-1 H-indole	R11	130.15
2	6.247	37.30	Phenol, 2,4-bis (1,1-dimethylethyl)	R11	191.15
3	6.289	0.47	Phenol, 2,4-bis (1,1-dimethylethyl)-	R13	191.15
4	6.840	4.91	3-Octadecene, (E)-	R13	55.05
5	7.916	0.38	Benzoic acid, 2,4-dihydroxy-3,6-dimethyl-, methyl	R13	136.10
6	8.336	33.86	9-Eicosene, (E)-	R13	55.05
7	9.221	2.61	Hexadecanoic acid, methyl ester	R11	74.05
8	9.253	4.37	Hexadecanoic acid, methyl ester	R13	74.05
9	9.521	3.61	l-(+)-Ascorbic acid 2,6-dihexadecanoate	R13	43.10
10	9.590	1.98	Dibutyl phthalate	R13	149.05
11	9.696	29.59	3-Eicosene, (E)-	R11	43.10
12	10.383	4.00	10-Octadecenoic acid, methyl ester	R11	55.10
13	10.531	2.35	Hexadecanoic acid, 15-methyl-, methyl ester	R11	74.05

## Supplementary Materials

Supplementary File 1

## Abbreviations

PDA	Potato dextrose agar
CDA	Czapek Dox agar
MS	Murashige and Skoog
GC-MS	Gas chromatography and mass spectrometry

## References

[1] Dearanley J.W.D., Perotto S., Selosse M.-A., Martin F. (2016). Structure and development of orchid mycorrhizas. Molecular Mycorrhizal Symbiosis.

[2] Wilson D. (1995). Endophyte: The evolution of a term, and clarification of its use and definition. Oikos.

[3] Hardoim P.R., van Overbeek L.S., Berg G., Pirttilä A.M., Compant S., Campisano A., Döring M., Sessitsch A. (2015). The hidden world within plants: Ecological and evolutionary considerations for defining functioning of microbial endophytes. Microbiol. Mol. Biol. Rev..

[4] Tesitelova T., Tesitel J., Rihova G., Jersakova J., Selosse M.-A. (2012). Symbiotic germination capability of four Epipactis species (Orchidaceae) is broader than expected from adult ecology. Am. J. Bot..

[5] Rodriguez R.J., White J.F., Arnold A.E., Redman R.S. (2009). Fungal endophytes: Diversity and functional roles. New Phytol..

[6] Selosse M.A., Martos F., Perry B.A., Padamsee M., Roy M., Pailler T. (2010). Saprotrophic fungal mycorrhizal symbionts in achlorophyllous orchids: Finding treasures among the “molecular scraps”?. Plant Signal. Behav..

[7] Selosse M.-A., Schneider-Maunoury L., Martos F. (2018). Time to re-think fungal ecological niches?. New Phytol..

[8] Vujanovic V., Vujanovic J. (2007). Mycovitality and mycoheterotrophy: Where lies dormancy in terrestrial orchid and plants with minute seeds. Symbiosis.

[9] Vujanovic V., St-Arnaud M., Barabe D., Thibeault G. (2000). Viability testing of orchid seed and the promotion of colouration and germination. Ann. Bot..

[10] Zhang F.S., Lv Y.L., Zhao Y., Guo S.X. (2013). Promoting role of an endophyte on the growth and contents of kinsenosides and flavonoids of *Anoectochilus formosanus* Hayata, a rare and threatened medicinal Orchidaceae plant. J. Zhejiang Univ. Sci. B.

[11] Tsavkelova E.A., Cherdyntseva T.A., Botina S.G., Netrusov A.I. (2007). Bacteria associated with orchid roots and microbial production of auxin. Microbiol. Res..

[12] Cameron K.M., Chase M.W., Whitten W.M., Kores P.J., Jarrell D.C., Albert V.A., Goldman D.H. (1999). A phylogenetic analysis of the Orchidaceae: Evidence from rbcL nucleotide sequences. Am. J. Bot..

[13] Rokaya M.B., Raskoti B.B., Timsina B., Münzbergová Z. (2013). An annotated checklist of the orchids of Nepal. Nordic J. Bot..

[14] Rajbhandari K.R. Orchids of Nepal: Status, threat and conservation. Proceedings of the National Workshop on NTFP/maps Sector Action Plant Development: Orchid, Department of Plant Resources, Ministry of Forest and Soil Conservation and Central Department of Botany, Tribhuvan University.

[15] Xing Y.-M., Chen J., Cui J.-L., Chen X.-M., Guo S.-X. (2011). Antimicrobial Activity and Biodiversity of Endophytic Fungi in *Dendrobium devonianum* and *Dendrobium thyrsiflorum* from Vietnam. Curr. Microbiol..

[16] Pant B., Paudel M., Chand M.B., Wagner S.H. (2016). Treasure Troves of Orchids in Central Nepal.

[17] Baek J.M., Kim J.-Y., Ahn S.-J., Cheon Y.-H., Yang M., Oh J., Choi M.K. (2016). *Dendrobium moniliforme* Exerts Inhibitory Effects on Both Receptor Activator of Nuclear Factor Kappa-B Bone Erosion in Vivo. Molecules.

[18] Brick J.M., Bostock R.M., Silverstone S.E. (2004). Rapid in situ assay for indole acetic acid production by bacteria immobilized on nitrocellulose membrane. Appl. Environ. Microb..

[19] Pandey M., Sharma J., Taylor D.L., Yadon V.L. (2013). A narrowly endemic photosynthetic orchid is non-specific in its mycorrhizal associations. Mol. Ecol..

[20] Lofgren L.A., LeBlanc N.R., Certano A.K., Nachtigall J., LaBine K.M., Riddle J., Broz K., Dong Y., Bethan B., Kafer C.W. (2018). *Fusarium graminearum*: Pathogen or endophyte of North American grasses?. New Phytol..

[21] Gonzaga L.L., Costa L.E., Santos T.T., Araújo E.F., Queiroz M.V. (2015). Endophytic fungi from the genus Colletotrichum are abundant in the *Phaseolus vulgaris* and have high genetic diversity. J. Appl. Microbiol..

[22] Davis E.C., Franklin J.B., Shaw A.J., Vilgalys R. (2003). Endophytic *Xylaria* (Xylariaceae) among liverworts and angiosperms: Phylogenetics, distribution, and symbiosis. Am. J. Bot..

[23] Wang X., Radwan M.M., Taráwneh A.H., Gao J., Wedge D.E., Rosa L.H., Cutler H.G., Cutler S.J. (2013). Antifungal activity against plant pathogens of metabolites from the endophytic fungus *Cladosporium cladosporioides*. J. Agric. Food Chem..

[24] Hermosa R., Viterbo A., Chet I., Monte E. (2012). Plant-beneficial effects of Trichoderma and of its genes. Microbiology.

[25] Narayan O.P., Verma N., Singh A.K., Oelmüller R., Kumar M., Prasad D., Kapoor R., Dua M., Johri A.K. (2017). Antioxidant enzymes in chickpea colonized by *Piriformospora indica* participate in defense against the pathogen Botrytis cinerea. Sci. Rep..

[26] Dolatabad H.K., Goltapeh E.M., Safari M., Golafaie T.P. (2017). Potential effect of *Piriformospora indica* on plant growth and essential oil yield in *Mentha piperita*. Plant Pathol. Quarantine.

[27] Mandal S.M., Chakraborty D., Dey S. (2010). Phenolic acids act as signaling molecules in plant-microbe symbioses. Plant Signal. Behav..

[28] Baltruschat H., Fodor J., Harrach B.D., Niemczyk E., Barna B., Gullner G., Janeczko A., Kogel K.-H., Schäfer P., Schwarczinger I. (2008). Salt tolerance of barley induced by the root endophyte *Piriformospora indica* is associated with a strong increase in antioxidants. New Phytol..

[29] Robinson M., Riov J., Sharon A. (1998). Indole-3-Acetic Acid Biosynthesis in *Colletotrichum gloeosporioides* f. sp. aeschynomene. Appl. Environ. Microbiol..

[30] Peters N.K., Verma D.P.S. (1990). Phenolic compounds as regulators of gene expression in plant-microbe interactions. Mol. Plant-Microbe Interact..

[31] Gallie D.R. (2013). L-Ascorbic Acid: A Multifunctional Molecule Supporting Plant Growth and Development. Scientifica.

[32] Noctor G., Veljovic-Jovanovic S., Foyer C.H., Grace S. (2000). Peroxide processing in photosynthesis: Antioxidant coupling and redox signaling. Philos. Trans. R. Soc. B.

[33] Vandenkoornhuyse P., Quaiser A., Duhamel M., Van L., Dufresne A. (2015). The importance of the microbiome of the plant holobiont. New Phytol..

[34] Selosse M.-A., Bessis A., Pozo M.-J. (2014). Microbial priming of plant and animal immunity: Symbionts as developmental signals. Trends Microbiol..

[35] Khan A.L., Al-harrasi A., Al-rawahi A., Alzfarsi Z., Al A., Waqas M., Shin J. (2016). Endophytic fungi from frankincense tree improves host growth and produces extracellular enzymes and Indole Acetic Acid. PLoS ONE.

[36] Séne S., Avril R., Chaintreuil C., Geoffroy A., Ndiaye C., Dieddhiou A.G., Sadio O. (2015). Ectomycorrhizal fungal communities of *Coccoloba uvifera* L. mature trees and seedlings in the neotropical coastal forests of Guadeloupe (Lesser Antilles). Mycorrhizal.

[37] Murashige T. (1974). Plant propagation through tissue culture. Ann. Rev. Plant Physiol..

[38] Baldi A., Srivastava A.K., Bisaria V.S., Varma A., Kharkwal A.C. (2009). Fungal Elicitors for Enhanced Production of Secondary Metabolites in Plant Cell Suspension Cultures. Symbiotic Fungi.

[39] Parry C., Blonquist J.M., Bugbee B. (2014). The optical/absolute chlorophyll relationship. Plant Cell Environ..

