# Supplementary material for: Isolation and Characterization of Plant Growth-Promoting Endophytic Fungi from the Roots of Dendrobium moniliforme
Source: Plants (Basel). 2018 Dec 28;8(1):5. doi: 10.3390/plants8010005 (PMC6359427; doi:10.3390/plants8010005)
Supplement: Supplementary File 1 [file plants-08-00005-s001.pdf]

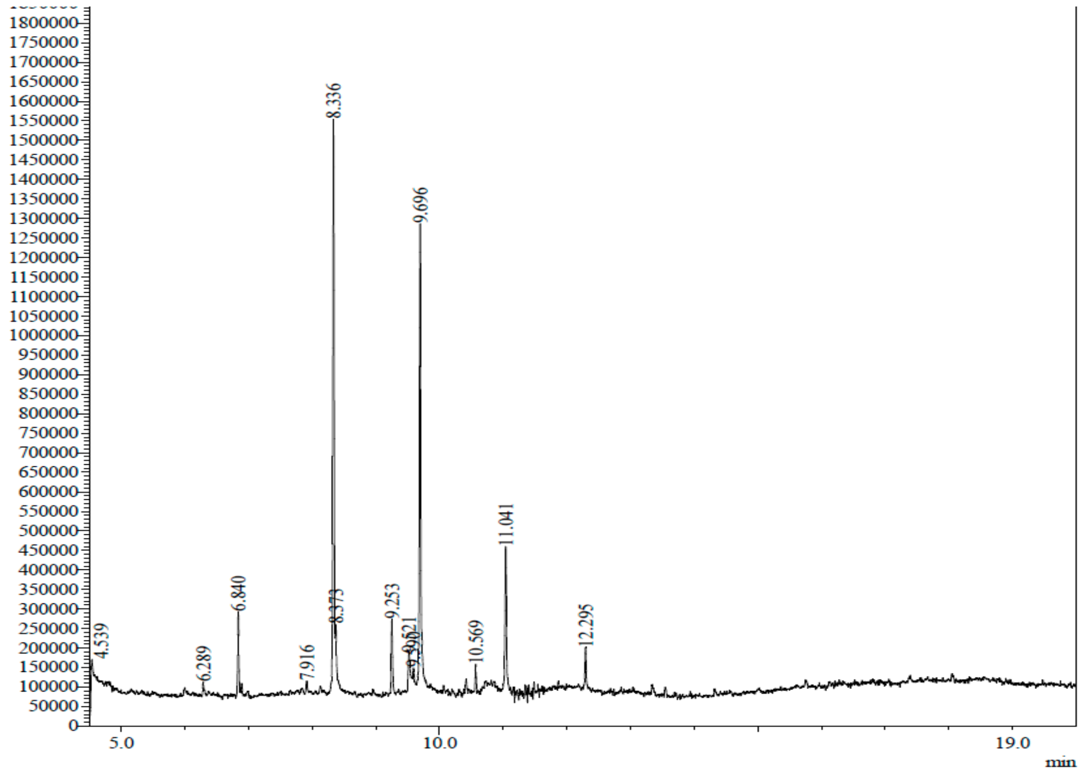

Figure1. Gas chromatography-mass spectrometer (GC–MS) analysis of the organic extract of fungus R13 (*Fusarium* sp.) Compounds are identified in the table 2.

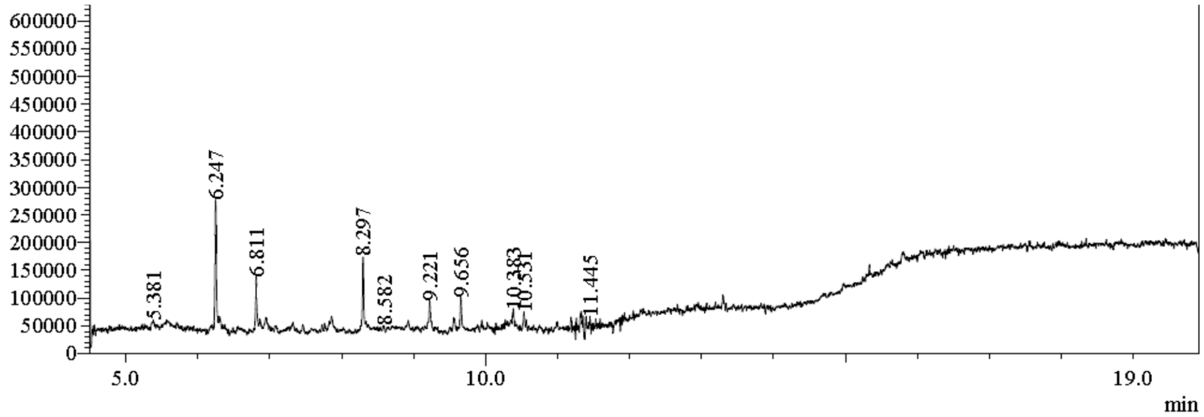

(A)

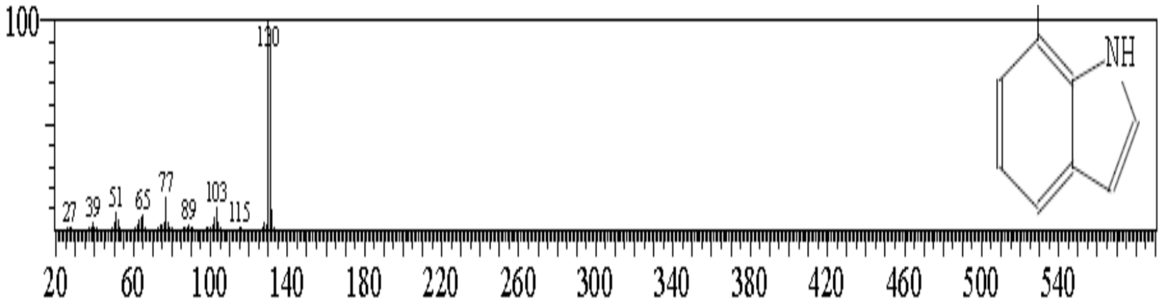

(B)

Figure 2. Gas chromatography-mass spectrometer (GC–MS) analysis (chromatograph) of the organic extract of fungus R11 (A) Compounds are identified in the table 2. The presence of IAA compound or its derivative in trace amount (B).
